# A study of the influence of dexamethasone on lipid profile and enzyme lactate dehydrogenase

**Published:** 2015

**Authors:** A Arab Dolatabadi, M Mahboubi

**Affiliations:** * Department of Biology, Payame Noor University, I.R. of Iran.; ** Abadan School of Medical Sciences, Abadan, Iran

**Keywords:** dexamethasone, lipid profile, lactate dehydrogenase, rat

## Abstract

Dexamethasone is an exceptionally applied glucocorticoid unfortunately prescribed too much. This drug is attached to its receptors in the cytoplasm by going through the cell layer, and opens the cell nucleus by the drug-receptor system, being ultimately responsible for systematic effects of corticosteroids. This study was conducted to explore the influence of dexamethasone on serum level of some biochemical parameters in adult men rats.

40 adult male rats were put into 4 test and check collections. The test collection only received saline and the experimental group received dexamethasone of 0.4, 0.7, and 1 mg/ kg doses daily in the form of intraperitoneal inoculation of 1 mL/ day. After serum separation, the serum value of cholesterol, triglycerides (TG), low-density lipoprotein (LDL), high-density lipoprotein (HDL) and lactate dehydrogenase were measured and the outcomes were examined by using SPSS and Dunnett software.

The test of lipid profile and lactate dehydrogenase was done by using the biochemistry tools and the collections were examined. In this research, meaningful differences in the application of the above hormones were not observed up to 0.7 mg/ kg dose. However, important differences were seen in higher doses i.e. 1mg/ kg in the test collection associated with the administration group (P<0.05).

The final result was that the injection of dexamethasone followed in the development of cholesterol and adverse lipid and it could cause tissue damage by increasing lactate dehydrogenase.

## Introduction

Corticosteroids refer to steroids that are generated in the cortical section of the adrenal gland. Due to the significant role of glucocorticoids in mitigating the immune responses, a high number of drugs have been made based on this structural skeleton with similar chemical formulas that are called steroid or corticosteroid. Corticosteroid drugs include betamethasone, dexamethasone, hydrocortisone, triamcinolone, methylprednisolone, prednisone, clobetasol, beclometasone, fludrocortisone, fluocinolone, fluticasone, etc. [**1**]. Dexamethasone ampoule is the most ordered drug between injectable drugs in Iran. Dexamethasone belongs to the group of synthetic corticosteroids that has important anti-inflammatory and anti-allergic influences and results in the pain of inflammatory processes, especially in joints. Also, dexamethasone outcomes in the destruction of the immune method, and these effects can often influence different systems of the body. Delaying the healing of wounds, affliction with diabetes, the effect on the balance of body fluids and electrolytes that results in retention of salt and water in the body, the effect on the distribution of lipids in the body and consequently the quantity of lipids in specific sections of the body such as back of the neck, increase of hypertension, blood sugar and excessive hairs in different parts of the body such as face, especially in females, being among the other adverse effects of inappropriate and excessive use of this ampule [**2**]. It should be remarked that systemic corticosteroids (dexamethasone) as an initial treatment for resolving simple and chronic allergies and the symptoms, could be controlled by drugs having less adverse effects, such as antihistamines. Dexamethasone can result in harmful influences, and one should visit the doctor if the signs of negative impacts do not disappear or continue for a long time. Its side effects include irritability and stomach pains, vomiting, headache, insomnia, depression, anxiety, acne and pimple, an increase of hair growth, irregular menstrual periods, weakening of the immune system, delay in wound healing, creation of complications such as hallucination and mental disorders and the emergence of maniac attacks, the influence on the balance of body fluids and electrolytes that outcomes in the maintenance of salt and water in the body, influence on the number of lipids in the body and consequently the accumulation of lipids in particular sections of the body such as back of the neck, increase of hypertension, blood sugar (hyperglycemia), diabetes and increase of blood lipids which are all known as active factors in the experience of cardiovascular illnesses and strokes [**3**]. The decrease in bone density, osteoporosis, and a higher dose of it results in tendon rupture or injury [**4**]. The increase of the pressure inside the eyeball and the occurrence of glaucoma particularly in old people and also the appearance of cataracts, reduction, and weakening of mucosal layers of the gastrointestinal tract especially stomach, which results in the emergence and aggravation of peptic ulcers. Glucocorticoids rapidly spread in the circulatory method and cause the direction of transcription of any genes when passing the cell membrane by attaching to cytoplasmic receptors. Glucocorticoids make the genes that have a significant function in infection such as cytokines and passionate enzymes such as nitric oxide synthase, inactive [**5**]. NO has a dual conflicting biological activity which means that has both cellular toxicity effects and protective effects [**6**]. NO has inherently cellular toxicity effects and participates in the formation of a strong oxidant such as pentoxi nitrite during a series of reactions with superoxide anion [**7**]. Dexamethasone is an extremely applied glucocorticoid unfortunately prescribed too much. This drug is attached to its receivers in the cytoplasm by crossing into cell membrane and opens the cell nucleus by the drug-receptor complex. By connecting to particular regions of DNA, this mixed outcomes in the stimulation of mRNA transcription and then the production of enzymes that are presently effective for natural results of corticosteroids. Dexamethasone uses its anti-inflammatory results by limiting the increase of inflammatory cells in the inflammation field, phagocytosis inhibition, and release of enzymes that are responsible for inflammation and interference of making and freedom of chemical mediators of inflammation [**7**]. The high level of triglycerides also grows the danger of metabolic syndrome. Cholesterol is a material made of lipid that belongs to a collection of lipids called steroids. Carrier molecules made of a protein called apoproteins are turned into lipoprotein when they are combined with cholesterol and triglycerides. The increase of its level enhances the chance of cardiovascular diseases. LDL deposits cholesterol on artery walls, which in turn, results in the form of a thick and hard material called plaque. Over time, this plaque becomes thicker and outcomes in the narrowing of blood vessels or the plaque, being ruptured and separated from the artery wall, which results in blood clotting and blockage of the artery in the place of rupture or the clot may be carried to other parts of the body; this process being called atherosclerosis. Cholesterol exists in tissues and plasma in the form of free cholesterol or in the kind of storage of it, which means attached to fatty acids or in a long chain in the shape of cholesterol ester. Cholesterol is an amphipathic lipid and is a basic fundamental component of membranes and outside layers of plasma lipoproteins. Cholesterol and ester cholesterol are carried to body tissues by low-density lipoprotein (LDL). During a process called reverse transfer, cholesterol is transported to the liver by high-density lipoprotein and is eliminated from the body there in the kind of free cholesterol or after turning into bile acids. Hyperlipidemia is an abnormally great level of blood lipid. Types of hyperlipidemia (types iv, iii, ii, I, and v) are defined based on the degree of lipid in the blood and their higher levels than the average level [**8**]. Therefore, considering the increase in the use of glucocorticoids, paying more attention to the opposing influences of these drugs is important, and thus the influence of dexamethasone on lactate dehydrogenase and lipid profile was explored in this research.

## Methods

**Devices**

Centrifuge machine (Eppendorf), AutoAnalyzer device. The biochemical analyses were performed by biochemical AutoAnalyzer BT plus 3000 made by the Italian company, Biotecnica that can do biochemical, immunology, serology, and drug level tests. Ketamine was applied for anesthetizing rats. The lab tools triglycerides, cholesterol, LDL, HDL, and lactate dehydrogenase have been bought from Company of Pars Azmoon that has a source laboratory confirmation.

## Materials

Mature men Wistar rats were applied for the tests. 40 rats aged eight weeks and weighting 250-350 g in for 10-membered groups (1 command collection and four experimental groups) were bought from Pasteur Institute of Iran. The rats were kept in polypropylene cages whose floor was covered with sawdust, and the cage had an appropriate water container in the established position at the heat of 22 ± 1⁰C and humidity of 60 ± 10% and light of 12 hours per day and 12 hours per night with access to water and complete food (concentrate). All the rats were placed into an animal nest in the similarly environmental positions for two weeks ago the tests so that they were accustomed to the environment regarding adaptation, familiarity, and diet. All the animal experiments were done based on the moral committee. The rats in any collection were specified by some marks and they were fed intraperitoneally for ten days.

**Measuring heat and wetness of the situation**

Temperature and humidity were controlled to provide a pleasant temperature and moisture in the position and to maintain the temperature at 22 ± 1⁰C and humidity at 60%.

**Taking blood from the rats and preparing serum**

After 10 days of injection, blood was taken to perform biochemical tests. Serum was divided by using a centrifuge device with 3000 RPM rotation for 10 minutes. Also, it was given to AutoAnalyzer device to estimate the frequency of LDH, TG, HDL, LDL and cholesterol and the concentrations of the enzymes were calculated according to to the international unit per liter. This device can be applied for biochemical and enzyme tests. This device has high effectiveness and can do 200 tests per hour.

**Statistical analysis**

The data obtained from AutoAnalyzer device was saved by utilizing SPSS application and then it was transferred to EXCELL and necessary edits were performed. Then, its parameters (tissue damage and the action of enzyme LDH) were extracted in the two methods of injection and contact and the data resulted from the ANOVA table was removed from the SPSS programs and were reported and used in SPSS. The outcomes were shown in the mean and standard deviation. Considering the normalness of data distribution, ANOVA tests with repeated measurements were applied for the analysis of the results of the enzyme in each group before and after the experiment. Moreover, ANOVA and Dunnett analyses were used to analyze the groups with each other in each period. Also, the frequency table was delineated for tissues. The significance level was considered lower than 0.05.

## Results

**Fig. 1:**
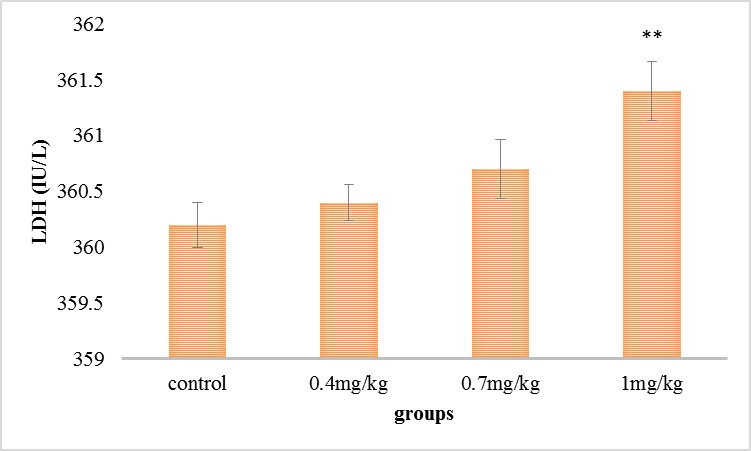
Comparison of the average of serum’s lactate dehydrogenase in the experimental groups

As it can be viewed in **Fig. 1**, the lactate dehydrogenase level was grown in all the test collections compared with the command collection, but this improvement was significant in the collection getting 1 mg/ kg.

**Fig. 2:**
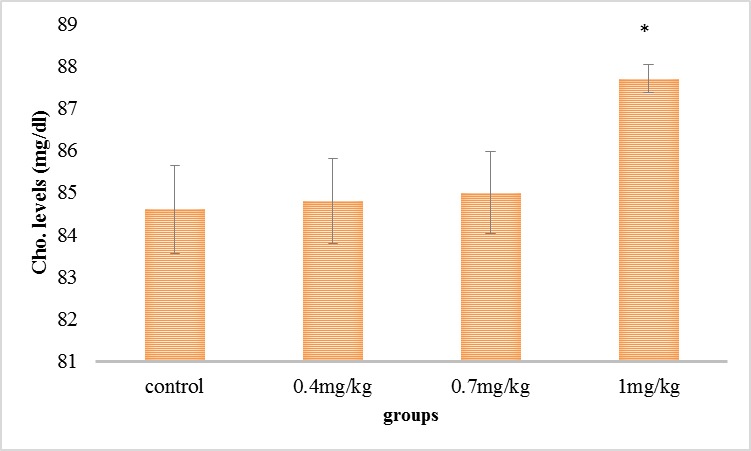
Comparison of the mean of the serum’s cholesterol in the experimental groups

As it can be viewed in **Fig. 2**, serum’s cholesterol level was raised in all the test collections associated with the command collection, but this increase was necessary in the collection getting 1mg/ kg.

**Fig. 3:**
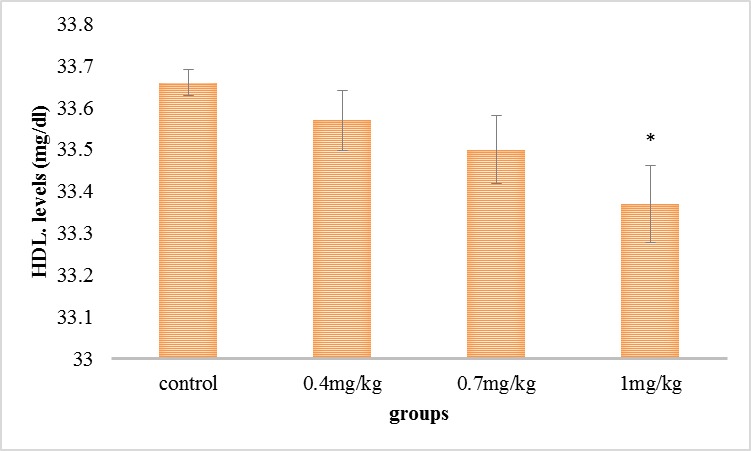
Comparison of the mean of the serum’s HDL in the experimental groups

As it can be seen in **Fig. 3**, the serum’s HDL level was reduced in all the experimental groups compared with the control group, but this reduction was significant in the group receiving 1 mg/ kg.

**Fig. 4:**
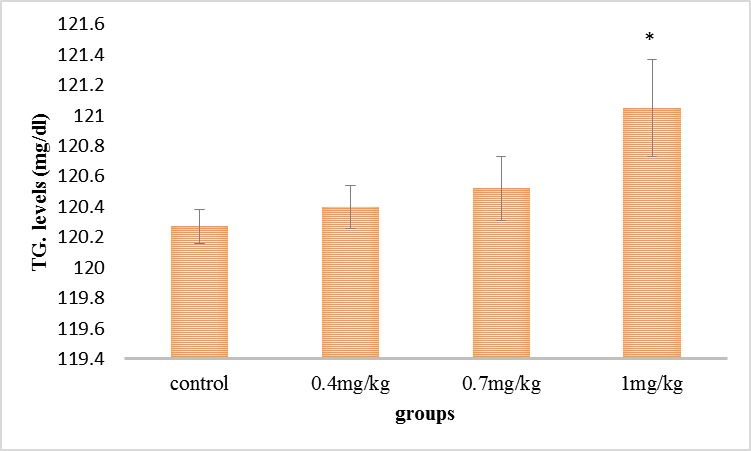
Comparison of the mean of the serum’s triglyceride in the experimental groups

As it can be seen in **Fig. 4**, the serum’s triglyceride step was grown in all the experimental groups compared with the control group, but this increase was significant in the group receiving 1 mg/ kg.

**Fig. 5:**
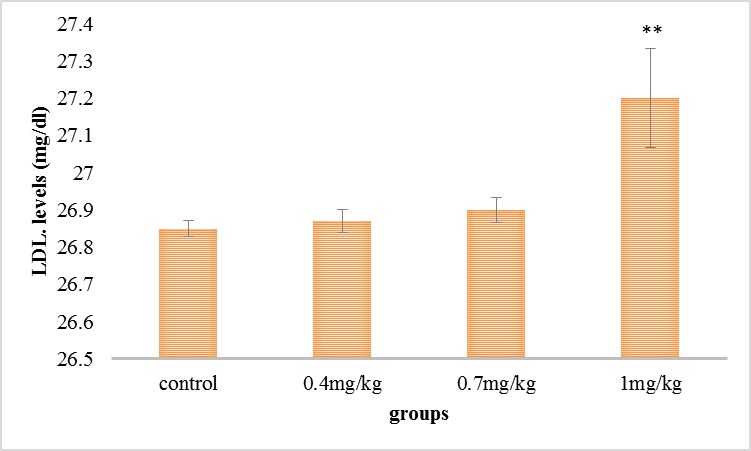
Comparison of the mean of the serum’s LDL in the test collections

As it can be seen in **Fig. 5**, the serum’s LDL step was grown in all the experimental groups compared with the control group, but this increase was significant in the group receiving 1 mg/ kg.

## Discussion

In this research, the influence of dexamethasone in different doses resulted in the reduction of HDL and the increase of CT, LDL, TG, and LDH and the changes were significant in high doses, which indicated a glucocorticoid impact lipid profile and flow of enzymes from body tissues. A study conducted in 2012, highlighted that the influence of the metabolic administration of glucocorticoid (dexamethasone) on plasma HDL and LDL and found out that dexamethasone results in a weak but significant increase in the body weight and increase of high-density lipoprotein and cholesterol but it did not have a major impact on triglyceride and VLDL-apoB [**9**]; which was compatible with the conclusions of the current research. Furthermore, in this research, plasma LDL level was raised, and the HDL level was decreased with the growth of the injective dose of dexamethasone. Glucocorticoids act as anti-inflammatory and immunosuppressant in patients with rheumatism and pulmonary diseases, which symbolizes that glucocorticoids result in the rise of HDL-cholesterol concentration [**10**]. since it was presented in **Fig. 2** and **3**, if dexamethasone is frequently injected, it can result in the development of total cholesterol and HDL of plasma, and cholesterol is similar to other lipids and oils and is not soluble in water (blood), being transferred in the blood by the aid of a particular type of protein named lipoproteins and mixture with them. After the absorption of intestines, both of the cholesterol and triglycerides are set in a protein cover called chylomicron. In fact, the group of triglycerides and cholesterol that are surrounded by Lipoprotein cover are called chylomicron, and 90% of it consists of triglycerides, and only 10% of it consists of cholesterol. Carrier molecules formed of proteins, called Apoproteins, are turned into lipoproteins when they are mixed with cholesterol and triglycerides which include LDL, HDL, and VLDL [**11**]. The tool accountable for the rise of glucocorticoid is due to the increase of HDL and HDL-cholesterol but most probably it will result in the decrease of cholesterol ester transferase protein and increase of the secretion of Apo- A-1 (the main protein of HDL particles) from liver [**12**]. In a study that was conducted by VrdoljokA (2015), the effect of dexamethasone on lipids and lipoproteins of plasma was explored, and the outcomes indicated that triglyceride, cholesterol, and HDL-cholesterol are raised, and the LDL cholesterol concentration is decreased [**13**]. In addition, in the present study too, exactly the levels of triglyceride, cholesterol and LDL were increased and the level of the serum’s HDLL was reduced, and, with the increase of dose, these changes were increased and they were statistically significant (P<0.05). LDL has been mentioned as a carrier of drug for specific places in different studies because LDL is incorporated in cells through the LDL receptor system [**14**]. In most studies, LDL acts as the carrier of anti-cancer drug to cancer cells because many cancer cells have more LDL receptors compared with natural cells. Asai et al. pointed out that in a laboratory model, dexamethasone prevents the incorporation of modified LDL in macrophages in vitro [**15**]. In a study by Mahendran (2005) on the impact of dexamethasone on lipoproteins, the outcomes indicated that treatment of dexamethasone results in the rise of the steps of triglycerides, cholesterol, and fatty acids in plasma and liver tissue. The level of phospholipids was increased in plasma but it was significantly decreased in liver tissue after the treatment of dexamethasone in the experimental group compared with the control group. The activities of lecithin cholesterol transferase and liver lipoprotein lipase were reduced after the administration of dexamethasone. The steps of HDL- triglyceride and HDL-cholesterol did not change, while the levels of LDL and VLDL were significantly grown. The levels of lipids were kept at the normal level [**16**]. In the present study too, dexamethasone appeared in significant changes in lipid profile. In a study that was conducted by Kumar (2001), the outcomes indicated that dexamethasone results in the increase of cholesterol and triglyceride levels [**17**], which is compatible with the conclusions of the current research. As it was presented in **Fig. 2** and **4**, in this current investigation, the serum levels of cholesterol and triglyceride were grown. The rise of cholesterol can result in fatty liver and consequently the steps of beta-hydroxysteroid butyrate, non-esterified fatty acids, the rate of non-esterified fatty acids to cholesterol, total bilirubin, aspartate aminotransferase, lactate dehydrogenase and bile acids will be upper than the normal levels [**18**]. Also in this study, as dexamethasone grew the degree of cholesterol in serum in rats, a greater degree of lactate dehydrogenase was produced in serum. One of the tissues generated by lactate dehydrogenase is liver tissue and hyperlipidemia can cause severe damages to the liver tissue and the result in the secretion of liver enzymes [**19**]. In this current research too, more lactate dehydrogenase entered the blood with the increase of cholesterol in 1 mg/ kg dose.

## Conclusion

The findings of the current research showed that the injection of dexamethasone results in the increase of cholesterol, low-density lipoprotein (LDL), triglyceride and reduction of high-density lipoprotein (HDL) of blood serum. And, as low-density lipoprotein (LDL) of plasma is the means by which cholesterol and ester cholesterol are carried to different tissues, during a process called reverse transfer, LDL free cholesterol of plasma is transferred to the liver from tissues to the liver and is eliminated from the body there in the kind of free cholesterol or after turning into bile acids and with the increase of cholesterol synthesis, a serious damage occurring to the liver tissue and the outcomes in the secretion of the liver enzyme. 

**Authors Contribution**

This research was carried out in collaboration with all writers and assistants.

**Conflict of Interest**

Authors declared that there is no conflict of interest.
